# Temporal depth in a coherent self and in depersonalization: theoretical model

**DOI:** 10.3389/fpsyg.2025.1585315

**Published:** 2025-09-05

**Authors:** Alexey Tolchinsky, Michael Levin, Chris Fields, Lancelot Da Costa, Rachael Murphy, Daniel Friedman, David Pincus

**Affiliations:** ^1^Professional Psychology Program, The George Washington University, Washington, DC, United States; ^2^Allen Discovery Center at Tufts University, Medford, MA, United States; ^3^Wyss Institute for Biologically Inspired Engineering at Harvard University, Boston, MA, United States; ^4^VERSES AI Research Lab, Los Angeles, CA, United States; ^5^Department of Psychiatry at Lehigh Valley Health Network in Bethlehem, Bethlehem, PA, United States; ^6^Active Inference Institute, Davis, CA, United States; ^7^University Hospitals of Cleveland, Case Western Reserve University, Cleveland, OH, United States

**Keywords:** dissociation, TAME, dynamical systems, temporal depth, self, depersonalization

## Abstract

Multiple theoretical models of dissociative experiences have been formulated over the last century. These theories are clinically useful; however, it remains unclear if common factors exist in various pathways leading to an onset of dissociations. In this paper we provide a framework for building an integrated, dynamical model of dissociative experiences. This framework combines a first-principles-based perspective with nonlinear dynamical systems, clinical, and neurobiological perspectives. We propose that a substantial change in the parameter we call “temporal depth” can be a common factor in dissociative episodes of any etiology, moreover, we consider such a change to have causal power. In the follow-up series of papers, we will provide empirical data supporting the collapse of temporal depth in various kinds of dissociative experiences, a computer simulation that would test this model’s computational components, and preliminary ideas for therapeutic applications.

There is no self in a given moment: the self is defined by persistence over time. (Mitchell, 2023)

## 1 Introduction

Dissociative disorders (DDs), including dissociative identity disorder (DID) and depersonalization derealization disorder (DPDR), are prevalent in clinical practice. Loewenstein (2018) summarized international epidemiological studies in North America, Europe, the Middle East, and Asia and reported that in clinical samples, including both inpatient and outpatient populations, the prevalence of DDs reached 46%. In a comprehensive review, Boyer et al. (2022) reported that the lifetime prevalence of DDs in the general population was estimated to be higher than that of bipolar disorder or obsessive-compulsive disorder. The International Society for the Study of Trauma and Dissociation (ISSTD), in their third version of the guidelines for the treatment of dissociative disorders, reported that DDs significantly impair patients’ functioning and present considerable risk – 67 percent of the patients diagnosed with DDs reported a history of repeated suicide attempts (International Society for the Study of Trauma and Dissociation, 2011).

Dissociative disorders are difficult to diagnose. Individuals with DDs, on average, spend from 5 to 12.4 years in some form of mental health treatment before receiving an accurate diagnosis (Boyer et al., 2022). Several reasons have been proposed to account for this, including the clinician’s difficulty in imagining this level of psychopathology, the patient’s lack of trust in disclosing awareness of their dissociative difficulties and the patient’s unawareness that they dissociate. When the diagnosis is reached, outpatient psychotherapy is typically recommended for DDs as the front-line treatment, while pharmacological treatments show marginal efficacy (International Society for the Study of Trauma and Dissociation, 2011).

Trauma-related etiological models of DDs appears to have stronger support among clinicians than alternative theories (International Society for the Study of Trauma and Dissociation, 2011). More specifically, prolonged elevation of stress accompanied by repeated traumatic experiences in circumstances where a person has no escape (e.g., chronic childhood abuse and neglect) are associated with dissociative conditions (reviewed in Lanius et al., 2018; see also Vonderlin et al., 2018). Clinicians refer to these circumstances as complex post-traumatic stress disorder (C-PTSD, see Herman, 2015), which has a different profile from one or several traumatic exposures, leading to the onset of post-traumatic symptoms (referred to as Acute PTSD). Indeed, approximately 90 percent of individuals with DID in the United States, Canada, and Europe experienced childhood abuse and neglect (American Psychiatric Association, 2022).

This article is the first one in a series of papers that present an integrated theoretical model of dissociative experiences. We hope in this series of papers to highlight one of the common factors that mediate an onset of dissociative symptoms in various etiological scenarios, such as psychological trauma, panic disorder, temporal lobe epilepsy, lesions in the brain, and the use of tetrahydrocannabinol (THC) or ketamine. We suggest that despite the important differences in various causes leading to the onset of dissociative symptoms, there is likely a common pathway where various kinds of pathogenesis converge.

The latest International Society for the Study of Trauma and Dissociation (2011) recommendations for psychotherapy of patients with DDs suggest that “treatment should move the patient toward better integrated functioning whenever possible (p. 132).” Our view is that by “better integrated functioning” they refer to the better integration of the patient’s Self. We see the Self as a process in time and a coherent linkage of the Self through time is related to the core concept of our paper, the “temporal depth,” which represents how far into the future the agent can plan and how far from the past it can recall. A collapse of the temporal depth may lead an agent to living in the “here and now”^1^ accompanied by the inability to access knowledge of the past or plan for the future. We propose that restoring the patient’s temporal depth is a common prerequisite for the stability, coherence, and continuity of the Self.

An additional component of our model as applied to psychotherapy is that both integration and disintegration are necessary at different times during the therapeutic process. We suggest that for the patient who experiences persistent dissociative symptoms, some features of DID or DPDR become relatively stable. A shift from these maladaptive regimes toward a more integrated, coherent Self implies a de-stabilization of the maladaptive regime (technically, an attractor landscape) corresponding to DID or DPDR and subsequent stabilization of an alternative ‘healthy functioning’ regime. In other words, while the long-term therapy goal should be the improved stability of the integrated, coherent Self, getting there may require a change, which is a destabilization of the maladaptive dynamics.

We find it useful to contextualize our proposal in the diverse literature on dissociative experiences that accumulated over the course of a century. Ludovic Dugas, who coined the term ‘depersonalization’ in 1898, was studying the psychopathology of “false memories,” including déjà vu (Sierra, 2009). Thus, phenomenology, the patient’s subjective experiences, was the original method of inquiry. Subsequently, many theoretical models of depersonalization and derealization were developed, including theories implicating the sensory systems, memory, affect, etc. (see Sierra and Berrios, 1997 for review).

Some of the current theories of depersonalization, derealization, and dissociative amnesia (Deane et al., 2020; Ciaunica et al., 2022) employ a top-down approach, where these dissociative states were modeled based on first principles, such as the Free Energy Principle (FEP, see Parr et al., 2022 for review). Other researchers chose a bottom-up approach, aiming to find the underlying mechanisms and structures of dissociative symptoms (Murphy, 2023; Lanius et al., 2018). In addition, clinicians working for decades with patients suffering from chronic dissociations share valuable qualitative observations, which add the richness of the patient’s subjective data to the abstract theoretical models (Chefetz, 2015).

Such diversity of viewpoints is clearly appropriate for the level of complexity in dissociative experiences. However, one of the challenges related to this multitude of models is that the authors from various disciplines use different terminology and methods of research and no current theory seems to coherently integrate phenomenology, dynamics, neurobiology, and other relevant perspectives. Psychotherapists are often reluctant to read papers with differential equations, such as those routinely used in the FEP articles (e.g., Friston et al., 2023). Similarly, some academic psychologists are less familiar with the clinical setting. Clinicians are justifiably concerned when researchers who have no clinical experience opine on how to best help the patients in psychotherapy (Shedler, 2006). Researchers, on the other hand, justifiably state that qualitative clinical case reports are useful, but often not sufficient to formulate the causal models of the clinical phenomena; and such reports can be augmented with falsifiable hypotheses, rigorous testing, etc.

We think that all these viewpoints usefully complement each other. Clinicians are correct that the abstract models of dissociative experiences lose the essential qualia. Consider the experience of one of Chefetz’s (2015) patients: “At one point I picked up the phone, was talking to my boss [while typing], and saw the words come out of my hands onto the computer screen, but they did not hit my brain and I had no idea what was going on. (p.125)” Can these subjective experiences be captured in mathematics or neurobiology?

The abstract models of dissociative experiences necessarily coarse grain the subjective human experiences. Such models help us see patterns and make testable predictions.^2^ However, this process comes at the cost of losing some of the depth of phenomenology.

An additional issue leading to the possible miscommunications between various theorists and practitioners is the heterogeneity of dissociative experiences. As an example, some clinicians suggest that affective flattening is an essential characteristic feature of dissociative disorders. However, they do not mention that post-traumatic flashbacks, which are also a kind of dissociation, are often accompanied by intense feelings, such as helplessness, pain, or rage.

We acknowledge the heterogeneity, which stands in contrast to drawing the bright lines in the definitions of depersonalization, derealization, and dissociative amnesia – separating some of them as the “true kind” of dissociative experiences. In agreement with Chefetz (2015), we take an approach of seeing dissociative experiences as heterogeneous and gradual, ranging from common, benign dissociative experiences to more severe, maladaptive forms of dissociative symptoms in DID or DPDR.

We hope in this and following papers to provide a possible interface for the collaboration of various disciplines involved and offer a model that attempts to integrate these perspectives. This model will necessarily be described in broad strokes as a preliminary framework. We will start by describing how we view a coherent and continuous mental/subjective Self from an information-theoretic perspective, including the Technological Approach to Mind Everywhere (TAME, Levin, 2022) and the FEP (Parr et al., 2022). We will then discuss dissociations from the dynamical systems perspective, as well as from contemporary neurobiological and clinical perspectives.

An important contribution of our model is to highlight the role of temporal depth collapse in dissociative experiences. A possible relationship between temporal depth and depersonalization has been previously suggested (Deane et al., 2020).^3^ Moreover, Friston (2018) wrote extensively on temporal depth being a necessary component underlying self-consciousness. In our paper, we would like to extend this hypothesis further, to a causal relationship. We suggest that a functional collapse in temporal depth leads to dissociative experiences, including depersonalization.

To clarify, we think that a collapse in a temporal depth can be an intermediate step in the chain of events leading to dissociative experiences; it is unlikely to be an ‘original’ or the only cause. For example, a problem with the functioning of the person’s episodic memory system can lead to the temporal depth collapse and dissociative experiences. We claim, based on the theoretical considerations to be developed below, that a dissociative experience reliably follows a temporal depth collapse, and a collapse of a temporal depth will reliably lead to a dissociative experience. We propose, in other words, that temporal depth collapse and dissociative experiences are highly correlated, with the former preceding and playing a causal role in the latter.

Formally testing the temporal depth collapse leading to an onset of a dissociative episode in humans would require an experiment. We have not identified non-invasive methods of temporarily and harmlessly reducing temporal depth in humans while keeping other relevant functions intact. In macaques, cryogenic deactivation technology has been used to temporarily deactivate dorsolateral prefrontal cortex (dlPFC) and other brain regions (Chan et al., 2015). However, we do not have a reliable way of assessing dissociative experiences in macaques.

In the absence of the experimental design to prove or falsify our hypothesis for humans, we are left with the analysis of the literature where temporal depth collapse and dissociative experiences co-occur. These data are correlational and serve as an indirect illustration of our model’s main thesis. In the follow-up papers we will present: (a) the empirical data showing this correlation in patients experiencing various kinds of dissociative experiences; (b) an analysis of currently used psychotherapeutic measures that we think influence the changes in the patient’s temporal depth; (c) a computer simulation that would test our model’s computational components.

Subsequently, should the computer simulation results support our hypothesis, we are hopeful to conduct additional studies with human subjects evaluating a relationship between temporal depth collapse and dissociative experiences. Such studies have already been done with individuals exposed to THC in laboratory settings (Melges et al., 1970; Mathew et al., 1993) and DPDR (Simeon et al., 2007). We hope to extend this work to an investigation of temporal depth variations in patients suffering from DDs before and after a comprehensive course of psychotherapy, such as psychotherapy incorporating Finding Solid Ground framework (Brand et al., 2022).

The measures used in these prior studies included a questionnaire called Temporal Integration Inventory (TII, Melges et al., 1970) a cognitive test evaluating temporal integration called Goal-Directed Serial Alternation (GDSA, Melges et al., 1970), one of the standard questionnaires evaluating dissociative symptoms, called the Dissociative Experience Scale (DES, Bernstein and Putnam, 1986), and positron emission tomography (PET) scanning.

We are open to a possibility that adding the measures of temporal integration/disintegration, such as TII and GDSA, to the evaluations of patients with DDs undergoing psychotherapy may lead to a more nuanced version of our hypothesis as applied to these cases, such as the hypothesis formulated by Simeon et al. (2007) that the relationship between temporal depth and dissociative experiences is mediated by a parameter called “absorption.” Absorption is defined as “the use of one’s full commitment of available perceptual, motoric, imaginative, and ideational resources to a unified representation of the attentional object” (Tellegen and Atkinson, 1974). When absorption scores are sufficiently high, the changes in the person’s consciousness are such that he/she perceives nearly everything, including time, as a part of a “fantasy world.” A person experiencing persistent dissociative symptoms and scoring high on absorption could perceive their past, their dreams, or television shows as reality.^4^

In what follows, we will describe the key concepts from the theoretical frameworks we appeal to in this paper, we will then use these theories to formulate an information-theoretic model of the Self experienced by an agent in health and pathology. These parts of the paper (Sections 2.1–3.3) are dense in computational and mathematical terminology. We provided a Glossary and illustrations for the key terms; however, this may be insufficient for the clinical audience less fluent with the computational frameworks. Reaching clinicians is highly important for us, as clinical efficacy is the primary goal of our efforts. Therefore, starting from Section 3.3 we included the clinical practice and neurobiological perspectives on dissociative symptoms and temporal depth, and we will provide less computationally dense materials in the subsequent papers.^5^

## 2 TAME and FEP as modeling frameworks

### 2.1 Technological approach to mind everywhere (TAME)

The emerging field of Diverse Intelligence seeks to develop rigorous frameworks for understanding and relating to unconventional minds (Baluška and Levin, 2016; Orive et al., 2019; Pio-Lopez, 2021). This ranges from biologically non-neurotypical humans to the impending plethora of altered, chimeric, and extended beings whose presence will explode outdated binary categories of orgainsm vs. machine (Clawson and Levin, 2022; Rouleau and Levin, 2023). One such framework is TAME (Levin, 2022), which is grounded in the biological principles governing the self-assembly of bodies and minds from cells during embryogenesis (Levin, 2019) and the fragmentation of emergent wholes by failure modes such as the morphogenetic dissociation disorder we call cancer (Levin, 2021).

The TAME framework is based on three foundational principles: (a) a commitment to gradualism; (b) an absence of privileged material substrates (material independence); and (c) a commitment to an empirical approach to research questions as compared to a philosophical debate in the absence of empirical data. The first principle suggests that there are no bright lines separating various organisms in terms of the complexity of their minds; instead, there is a gradual accumulation of complexity and organization. The second principle suggests that minds are not exclusive to neuron-based systems, or computer hardware-based systems; there is no privileged material that is necessary for the specific kinds of a mind to operate. The third principle suggests that experimental data, rather than opinions or conventions, are the appropriate standard of deciding on how intelligent a system is and how much agency it has.

**Figure 1:**
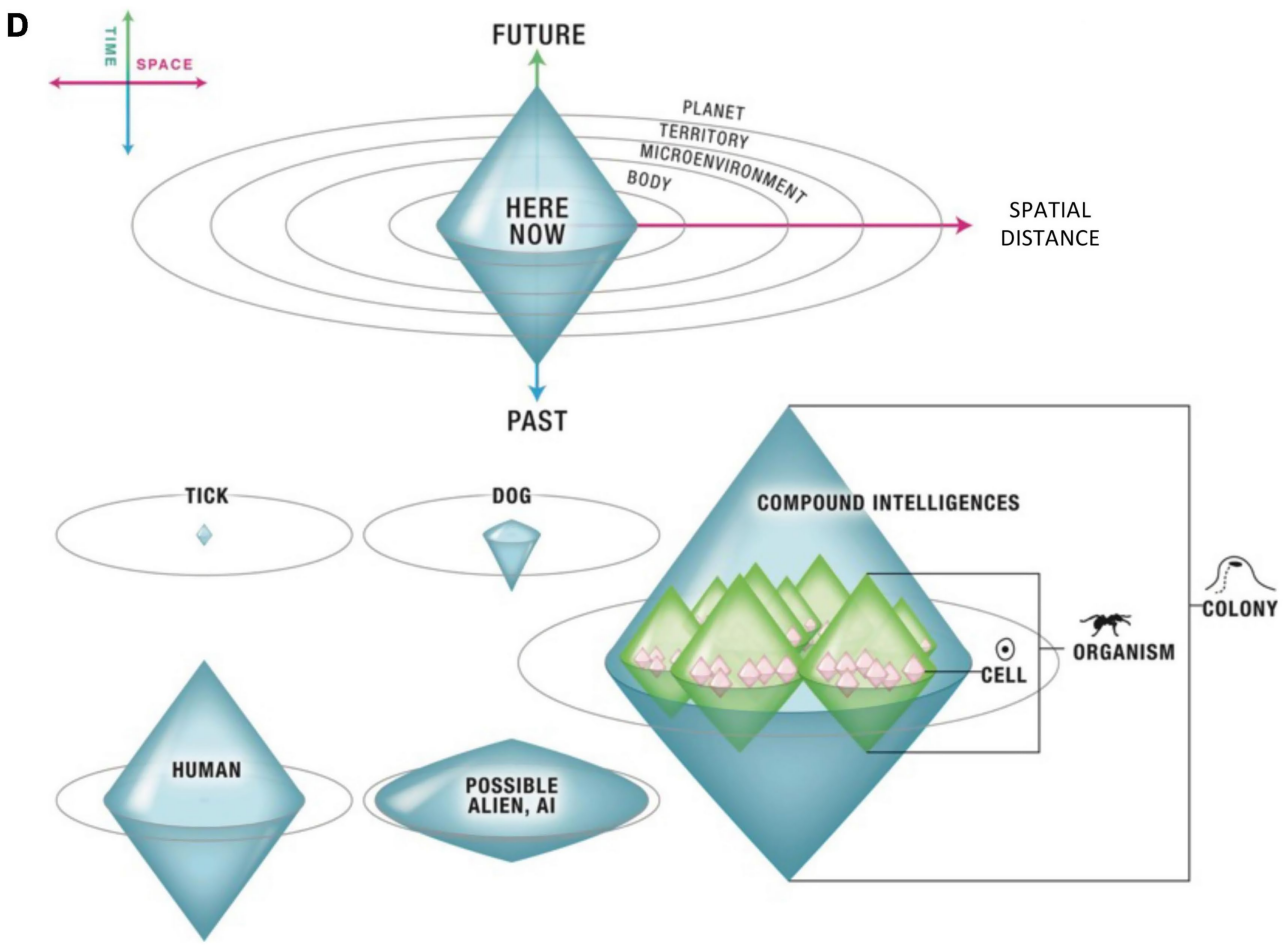
TAME cognitive light cone in agents ranging from a single cell organism to humans. TAME light cone is a measure of the biggest goal that an agent can pursue in space and in time. Reprinted with permission from Levin (2022).

One of the key concepts of TAME for our paper is the cognitive light cone, which is schematically depicted below on Figure 1. This concept captures the scale of an agent’s ability to use the past experiences to inform its present actions and to plan into the future, as well as the scale of its spatial goals. Put differently, TAME light cone is a measure of the biggest goal that an agent can pursue in space and in time. As you can see in the diagram, a tick operates in its immediate spatial environment and has very limited planning ability or memory of the past. A dog has a larger TAME light cone – it can travel further and can recall and plan more. Humans can support huge cognitive light cones that span the globe and have a time horizon known to be longer than their possible life span.

The size of the TAME light cone on the vertical axis is related to the focal concept in our paper – temporal depth. You can also see compound intelligences on Figure 1, including both a collective of cells - an organism and the collectives of animals, such as an ant colony. Under TAME, all intelligences are collective. An important feature of the TAME light cone concept with respect to the compound intelligences is emergence. An ant cannot build bridges, while an ant colony collectively can accomplish this; and a collective of cells can navigate a maze (Blackiston et al., 2021). What this implies is that the compound Self is not reducible to its components, a whole Self is greater than the sum of its parts, which is another way of saying that the compound Self is a non-linear system^6^ (see Rosas et al., 2024 for a technical treatment of the notion of “emergence” implied here).

Levin’s (2021) proposed model of a possible etiological pathway to cancer as the result of breakdown in communications between the adjacent cells is, perhaps, one of the most relevant examples of TAME framework applied to the concept of dissociation. Specifically, the closing of the gap junctions (intercellular connections that allow passage of small molecules) of one cell leads to it perceiving the rest of the cells as “not me” or “the environment.” This, in turn, leads to this newly isolated cell treating the environment as a food source; this cell also reproduces leading to metastasis.

The breakdown in the communications between cells effectively led to the fragmentation of the cell collective into two parts – the isolated cell, and the collective of cells without it. Should there be a closure of the gap junction in yet another cell in the remaining cell collective, that would in turn lead to further fragmentation into more entities. When one cell becomes informationally isolated from its neighbors, the previous cell collective’s cognitive light cone fragments into several smaller ones, leading to the temporal depth collapse. Therefore, the breakdown in communications between the components leads to both the spatial fragmentation and the loss of temporal continuity.

In the following section, we will show how the TAME cognitive light cone, its fragmentation and temporal depth collapse are equivalently described in the Free Energy Principle (FEP) framework.

### 2.2 Free energy principle

The free energy principle (FEP) was formulated by Karl Friston in the 2000s as a mathematical theory in neurobiology and extended thereafter to a general theory of living systems (Friston, 2013). One of the key ideas in FEP is that any system that persists will act to maintain its distinction from its environment. Stated more formally, Ramstead (2023) summarized one of the primary FEP claims as follows: “The free energy principle (FEP) says that if the generative model (or dependence structure) of a random dynamical system contains a Markov blanket (a conceptual boundary between the inside and the outside), then it will look as if internal states track the statistics of external states across the boundary.”

**Figure 2:**
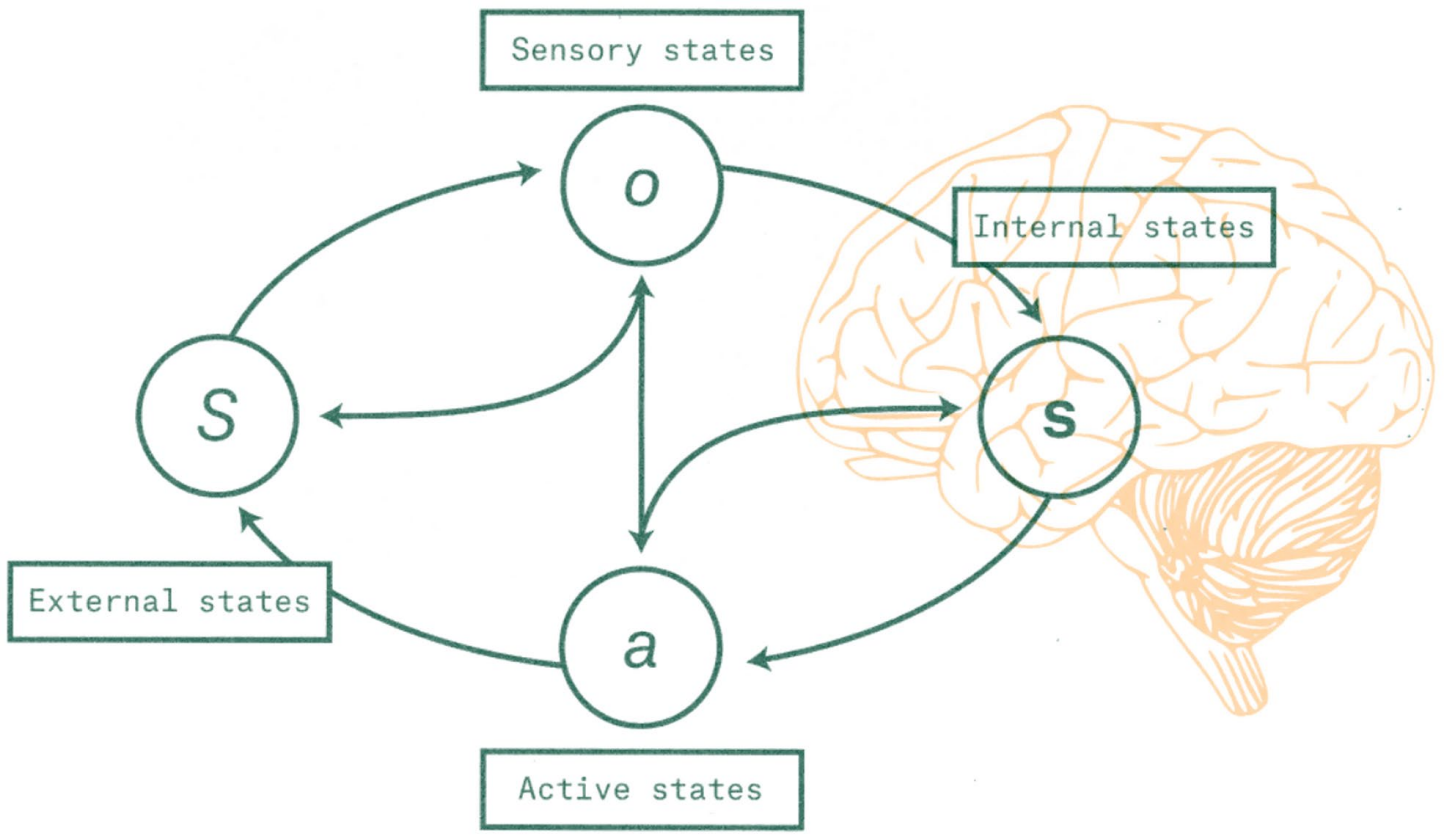
Structure of a Markov blanket as described by the FEP. Formally, a Markov blanket is a set of “boundary” states that separate the “internal” states of some system of interest – here, a brain – from the states of its environment. All interactions between the two must pass through, and hence be mediated by, the Markov blanket. Reprinted with permission from Ramstead (2023).

The Markov blanket is depicted on Figure 2. In a biological organism it is assumed to be composed of Active states and Sensory states.

To highlight what is pertinent for our paper, FEP suggests that a system that maintains its existence in the environment necessarily models this environment. This model generates predictions of inputs from the environment, so is termed a “generative model”; one can think of it as encoding the agent’s beliefs about how external states cause its sensory states. For example, if an agent senses warmth, it can infer probabilistically that the sun is shining on it. These models of the world are inferred from and hence adapted to the environment as it changes.

Some systems described in the classical FEP do not have Active States (e.g., a rock). In the presence of active states, the agent is also capable of model-driven action on the environment, and it does so in such a way as to introduce environmental changes in order to match the environment to the agent’s model (i.e., Active Inference: Parr et al., 2022). For example, a human has an internal belief that she can breathe; should she find herself immersed in water for more than a minute, she will attempt to get back to the surface to reduce the discrepancy between her model of being able to breathe air and the environment.

The generative models under FEP can have variable depth (see figure 12 in Friston et al., 2023). Agents with limited memory are only capable of modelling their immediate environment and live in the “here and now,” while agents with sufficient memory and processing power are capable of planning actions into the future. The generative models capable of planning are referred to as “temporally deep generative models.” The temporal depth introduced above can be formally described under the FEP as the length of the temporal horizon that is considered during planning, where planning entails counterfactually evaluating the consequences of future courses of action (Friston, 2018).

## 3 A model of the subjective (mental) Self^7^

### 3.1 TAME and FEP perspectives

#### 3.1.1 Material independent and belief-based

We see the Self as a component of a system’s generative model that can be implemented by systems of diverse material and structure. The Self is constructed dynamically from an organism’s – indeed, any system’s – continual efforts at making sense of both its external environment and its internal milieu. Therefore, our model of the Self is not inherently based on the physiology of the human body, including the physiology of the Central Nervous System (CNS). The CNS is just one of the environments where such models can be implemented. As we apply our model to mammals, including humans, we will describe the specific aspect of our model, the Core Self (see *3.1.2* below), which is embodied and evolved to help mammals adapt to their habitats and problem-solve in novel environments. While closely related to the body of a specific animal and its natural environment, the specific implementation of the Core Self can also be described in abstract FEP terms, e.g., in Solms (2021).

In addition, we see the organism as a system encoding beliefs (technically, probability distributions) that predict its own states and those of its world. The Self depends upon an organism’s ability to infer, i.e., on its generative model. The organism implements a generative model, some components of which are beliefs about the organism’s environment and other components of which are beliefs about the organism itself. We call the latter components the “self-model,” as it is experienced by an agent, or the “Self.”

In the healthy state, the Self represents the organism as embedded in, receiving sensations from, and acting on its environment. This representation of embeddedness and connection can fail, corresponding, in this model, to the pathological state of derealization. In the healthy state, the self also represents the organism as an agent with particular sensory and action capabilities and a particular remembered past. This representation of agency and history can also fail, corresponding, in this model, to the pathology of depersonalization.

#### 3.1.2 Hierarchy, composite system, boundaries

As defined above, the Self is a composite, nested, and embedded functional system (please see Compound Intelligences of Figure 1 as an illustration). We also consider the Self to be a system consisting of hierarchically structured beliefs/inferences, which effectively makes it a dynamic, hierarchical generative model (Parr et al., 2022).

The Self, taken in its entirety, is informationally separated from its external environment by a boundary, a Markov blanket which makes the Self conditionally independent from the non-Self (Parr et al., 2022, p.43). This boundary allows the Self to have a degree of separation and autonomy from its environment. The Self boundary is not material, like skin, but it is an informational boundary through which the “Self” and the “not-Self,” its environment, interact. To clarify, the Self boundary is the organism’s model of its biological boundary. We emphasize that the Self, as we have defined it above, is a model, and is distinct from the system – e.g. the organism – that constructs and implements it.^8^ The organism’s physiological body is not part of its Self, though the organism’s *model* of its body is (in general) part of its Self. The Self is, quite literally, a “construction of the mind.”

This is an important point. Any Markov blanket is a boundary in state space, not in physical three-dimensional space. A Markov blanket exists within the causal network of systemic and environmental variables and their causal relationships. While some boundaries happen to be simultaneously informational and spatial (e.g., skin), the Self’s Markov blanket is just informational.^9^ The Self, being a model, is an informational structure; hence its environment is also an informational structure. The Self’s Markov blanket separates, and maintains the independence between, these informational structures.

Under FEP, the Markov blanket is what separates “the thing” from the “not-thing” (Parr et al., 2022). In order for “the thing” to persist in time as a unique entity, the boundary’s elements and processes must remain functional and satisfy the properties of a Markov blanket, i.e., it must maintain conditional statistical independence between the “thing” and its environment. The entity informationally demarcated by the Self’s Markov blanket is the Self; the blanket also acts as the interface from the Self to its environment. The Self models its informational environment, it ‘senses’ it though the sensory states and ‘acts upon’ it via the active states (Parr et al., 2022).

Various components of the Self are separated from each other by their own functional boundaries, also Markov blankets (see Parr et al., 2022, p.43 for a description of nested Markov blankets). Collectively, all these boundaries play an important role in the stability of the Self and its various components.

As a hierarchical system, the Self has the “Core Self” component at the informational center of the hierarchy, and other components represent more peripheral layers around the Core Self.^10^ In our model, the Core Self is the concept that was described by Panksepp and Biven (2012) in Chapter 11 of their book “The Archaeology of Mind: Neuroevolutionary Origins of Human Emotions.” In mammals, the essential characteristic of this Core Self is that it is affective. Panksepp and Biven postulated that this Core Self was nonreflexive (anoetic) and dominated by raw affective feelings, and constituted a part of the purely affective, Core form of consciousness (Solms and Turnbull, 2018).

We take the Solms (2021) view on these raw affective experiences as “felt uncertainty,” which is a FEP-based conceptualization. In Solms’ model certain organisms do not have affects, but rather inflexible innate reflexes, such as a reflex to approach food and to avoid danger. Affects present an evolutionary advantage to animals that have them. Affects allows an animal to “feel through” the novel problem while using a specific homeostatic mechanism as a guide.

For example, if an animal that had never experienced high heat before were to find itself in a hot place, it could use the internal feeling of the body temperature to guide its actions. The animal will feel better when moving closer to shade and worse when moving away from it. The further the animal’s body temperature is from the homeostatic settling point, the worse it feels. A return from the high body temperature to the settling point would be accompanied by a positive feeling of cooling off. When the body temperature returns to the settling point, the feeling of being hot disappears entirely. The system being at or near the settling point suggests that the biological need underlying this affect is met.

This affective mechanism allows an animal to problem solve in novel environments. An organism that has only innate reflexes and no affects is far less likely to survive in completely unexpected circumstances – it would not have an inner “compass” to guide its actions.

A collection of these affective functions that are necessary for the animal’s survival constitutes the Core Self. Then, the set of predictions in the Core Self is that all the life-sustaining affects will be at or near their settling points. This state of balance where all biological needs are met corresponds to a minimum in the organism’s Variational Free Energy (VFE) – a biologically optimal state. An activation of one of the affects indicates a departure from the VFE minimum, which is a prediction error.^11^

Let us now illustrate this concept of the Core Self in neurobiological terms, making concrete some of the abstract terms in the informational model described above. Panksepp and Biven suggested that in mammals, the subcortical structures, including but not limited to the upper brain stem and the periaqueductal grey (PAG), mediated the functionating of the Core Self subsystem. Consequently, the Core Self is thought to be present in decorticated cats and hydranencephalic human children (Solms, 2019) – it does not require a functional neocortex.

The ‘higher levels’ of the brain’s structure in humans, including the neocortex mediate the higher levels of both consciousness and the Self – for example, our abilities to reflect on our own mental states and report them to others, referred to as an ‘extended consciousness’ (Solms and Turnbull, 2018). Additionally, the neocortex allows humans to have object representations. Then, at the level of the Core Self, we can experience a raw, primitive, wordless, but qualitatively distinct forms of affect, the nonverbal subjective experience: “I feel like this” (e.g., I feel hunger). Solms (2021) suggested that at the level of Core consciousness, without words or images, we can still differentiate a state of hunger from pain – qualitatively and subjectively. To summarize, with the object representation absent, the agent can still experience a raw form of a specific affective distress and then attempt to execute the behaviors to alleviate this distress.

However, with the higher levels of consciousness present, we can bind an objectless feeling to an object, as Solms (2021) describes it: “I feel like this about that (p. 204).” An example of such extension could be “I want an apple.” Meta-observations about oneself also rely on object representations. Therefore, observations such as “I look pale” or “I am a pessimist” are various forms of meta-cognition, where the “I” is a recognized mental object being reflected on, described, and thought about.

Let us reiterate this important point, “I” is a meta-cognitive construct, an abstraction, it only exists at the higher levels of the Self (e.g., in Autobiographical Self). It is not used, nor is needed in the Core Self. The Core Self, as a system, has the capacity to detect the affective prediction errors and attempt to minimize the VFE through action without any “I.” A meta-cognitive “I” is therefore an illusion in a sense of it not being a concrete object in the world; it is an abstract concept used in language and other forms of meta-cognitive processing (Metzinger, 2004; Seth, 2021; Graziano and Webb, 2015).

One of the reasons Solms and Turnbull (2018) labeled the fundamental, elementary form of consciousness “Core” is because of an asymmetry – the higher levels of consciousness cannot be functional without the Core, while the reverse is not true (Solms, 2021). Solms illustrated this statement with an example from Fischer et al. (2016) that a two-cubic-millimeter size lesion in the parabrachial nucleus reliably induces a coma, while no lesion that size anywhere in the neocortex would cause a cessation of consciousness.

The same can be said about the Core Self with respect to other Self components. Peripheral Self components cannot function without the operational Core Self, which effectively creates a hierarchical structure. In addition, as stated in Section 3.2, the Core Self can change the regime of functioning in other Self components by inducing phase transitions. Anatomically, in mammals, this corresponds to the regions of the brain participating in the Core Self functionality influencing the states of the cortical and subcortical brain structures through generalized arousal. We agree with Solms (2021) that the regions in the upper brain stem, including the Reticular Activating System constitute the area upon which consciousness depends; it is the source of arousal and, therefore, of consciousness, without it, no conscious activity (including the Self) is possible.

At the more informationally peripheral levels of the hierarchy, the Self is a composite system containing (a) a Bodily Self (Seth, 2021), an Autobiographical Self,^12^ a Social Self (Seth, 2021), and other components;^13^ and in which (b) each Self component has its own boundary. The Bodily Self refers to a system (generative model) dynamically building inferences about our body, including the various representations and re-representations of the bodily components, interoceptive processing, etc. An Autobiographical Self is a system dynamically representing our life’s history. This system relies on both the contextualized event memory (episodic) and the generalized, factual memory (semantic) in humans. A Social Self is a system representing our inferences about how we are seen by others and how we present ourselves and act in the social environment. Each of these components is embedded into the whole Self and it also contains sub-components, creating a nested architecture, as depicted on Figure 1.

The non-Core components of the Self are interrelated and influence each other. However, each component can experience a level of dysfunction while the remaining components remain reasonably operational. For example, some level of dysregulation in the Autobiographical Self can be accompanied by an intact functioning of the Bodily Self and vice versa. Thus, a hierarchical, composite structure makes the Self more resilient. With that, a serious dysfunction in the Core Self would lead to a total depersonalization–a complete loss of all aspects of the Self.

If we consider one of the Self’s components–the Autobiographical Self, or the Bodily Self, then a coherent and continuous, experienced “I am me” also implies that the current instance of the “I” in that subsystem is recognized as matching the representation of “me” encoded in the subsystem-specific memory. Conversely, a prediction error in “I am me” can be seen as an element of depersonalization. While nearly all the low-level components of the underlying physiological architecture (e.g., cells) are replaced throughout the person’s lifetime, the continuity of “I am me” is maintained at the level of a belief system, i.e., at the level of the Self as a constructed model.

Each component of the Self has an overall, unified ‘identity.’ For example, in a Bodily Self it would be ‘my body,’ which is a belief close to the core of the predictive hierarchy–“my body is coherent, persistent in time and it is all mine.” The ‘ownership’ is an important component of the bodily Self, and the ownership can also experience various forms of dysfunction. Additionally, due to the composite nature of each component, it will contain subcomponents, such as ‘my arm.’ The prediction errors related to each subcomponent vary in precision, ranging from nonpathological experiences in the rubber hand illusion and escalating to the disturbances that can be seen in somatoparaphrenia or body integrity disorder (BID). All these prediction errors are, among other things, forms of depersonalization. This phenomenon of ‘partial’ depersonalization can scale up to an out of body experience (OOB), where the entire body is seen separately as an object.

#### 3.1.3 The Self is experienced as a monadic whole while being a form of a collective intelligence

According to TAME, all intelligences are collective, while the Self is subjectively experienced by humans as a monadic “whole.” One aspect of this seeming contradiction could be the difference in perspectives - the collective intelligence view is usually the perspective of an outside observer, while the monadic Self is the perspective from within. However, even from this internal perspective, it is not obvious how the coherence of the Self is established and maintained. In our model, the tentative answer to this question is multifactorial, while we realize that it is incomplete.^14^

Seth (2021) shared a viewpoint on a monadic Self as a form of a “delusion” in a sense that it exists only at the level of a subjective belief and not in objective (outside) reality. We agree. Specifically, as applied to humans, we believe that the higher levels of the Self, such as the Autobiographical Self, create an impression of a unified experience, but this is just the experience of the Autobiographical Self and not of the entire system. The Autobiographical Self ‘claims,’ to itself and others, to be the entire Self, while it is not. Then, it is this meta system that is deluded because it “believes its own reports.”

Stated differently, we suggest that the presence of a stable belief “I am whole” in the Autobiographical Self’s generative model contributes to us feeling as a monadic Self. Thus, the subjectively perceived unity, the coherence of the Self is an inference.

A second component of the coherence of the Self is related to the informational scale of this phenomenon – the macro scale, as compared to the micro scale of individual neurons or meso-scale of neuronal ensembles. As we move up in the scale of investigation of the brain-mind phenomena, we tend to see the aggregation and coarse-graining of the data. For example, at the macro level of the scalp EEG we lose some data on the variability and noise happening at the micro-level. To illustrate this idea, we can move between the rooms in the house, however, from the standpoint of an observer standing outside, we remain in the same house – there is perception of higher stability/order at the higher scale of observation. This is another pathway of how the Self is experienced as monadic and coherent at the level of the self-conscious, metacognitive mind.

#### 3.1.4 Continuity of the Self in time: the assessment of familiarity/novelty

Another quality of the Self is its continuity in time. Similarly to the coherence, we suggest that the continuity of the Self in time is an inference. Nearly as a tautology stemming from the definition of a Markov Blanket, the Self will remain “the same” (persist in time) while all the processes/communications across its Markov blanket remain functional.

An additional component of the continuity of the Self and its various components in time is the experience of familiarity, the recognition of the Self to be familiar, not novel. This experience of familiarity can be described as a match, e.g., “I am the same now as I have been in the past.”

There is a long history of views on such calculations in neuroscience. As stated in Section 1, Dugas studied deja vu, which can be described as a temporary dysfunction of the ‘familiarity functional system,’ where something novel is perceived as familiar, while jamais vu can be seen as a dysfunction in another direction – where something familiar is perceived as novel. We could therefore describe one aspect of depersonalization as being similar to jamais vu – we perceive our body and mind as novel, unfamiliar.

Empirical data support the presence of the ‘familiarity functional systems’ as distinct from other kinds of memory systems; and a version of such familiarity assessment can be present for various mental functions (see Yonelinas et al., 2022 for review). For example, Meyer and Rust (2018) studied the visual recognition in monkeys and have demonstrated that there were dedicated, distributed, dynamical brain-mind systems (‘visual recognition memory’) that contributed to the familiarity calculations; and these systems were distinct from other aspects of visual perception. In a different domain of functioning, Darby et al. (2016) suggested that the retrosplenial cortex mediated the calculation of familiarity/novelty as part of the Capgras delusion in human subjects.

It may seem that the familiarity assessment may appear to be similar to a binary on/off switch. However, Meyer and Rust (2018) have shown that the predictive inference framework can indeed be used to perform such calculations and these calculations are probabilistic and not binary. Specifically, in Meyer and Rust’s view, a prediction error in expecting an object to be familiar constitutes the reaction of novelty; and such prediction errors can happen with variable degrees of precision, they are not all or nothing, even if they appear to us as such. The gradual nature of such calculations allows us to have the reactions such as: “you seem familiar, but I am not sure, did we meet somewhere before?”

To summarize, the familiarity/novelty aspect of depersonalization or derealization can be described by the FEP’s framework of Bayesian inferences, leading to the degree of familiarity being calculated as a continuous variable (Yonelinas et al., 2022). The subjective impression of us dichotomously perceiving something as familiar versus novel can be seen as the coarse graining of such continuous calculations, similar to a categorical perception of having a fever, while the underlying body temperature calculations are continuous.

#### 3.1.5 Representational capacity

The agent who infers must have a functional representational capacity for representing the world and itself (Da Costa et al., 2021) – a memory system. As noted earlier, the generative model of the Self and its components is hierarchical, or deep (Parr et al., 2022). This implies that the agent is capable of planning into the future, which, in turn, requires an ability to generate, store, and retrieve counterfactual data. When the representational capacity is completely impaired for any reason, the agent loses an ability to infer, leading to both severe depersonalization and derealization. A partial loss of representational capacity, e.g., in some form of amnesia, may lead to some loss of coherence or continuity in either the model of the inner milieu, the environment, or both.

#### 3.1.6 Healthy and pathological temporal depth changes

Healthy individuals are able to temporarily expand or contract temporal depth voluntarily to some degree through attentional control. Temporal depth is expanded during long-term planning, and is contracted during attention-demanding tasks, e.g., during “flow” states where successful performance is generally not self-conscious. During voluntary collapses of the temporal depth, an individual may experience a healthy hyper-focused state, e.g., “losing oneself” in a book, or in one’s lover’s eyes, or in meditation,

Involuntary collapses of temporal depth, however, are pathological, and may indicate a dysfunction in an underlying memory system. Should such memory dysfunction happen, an agent who had been capable of recalling the events of the remote past and planning far into the future would lose these abilities. The continuity of the Self, particularly the metacognitive Self, is partially or completely disrupted during such episodes, which is a feature of depersonalization. In these circumstances, the absorption experienced by these patients (as discussed in Section 1) may be an example of involuntary, pathological hyper-focused state.

It is important to highlight that some degree of depersonalization can be pursued voluntarily, e.g., in meditative practices (Deane et al., 2020) that employ intense concentration to temporarily “suspend” the metacognitive Self, and that when pursued voluntarily, it is not pathological and may even be therapeutic. These experiences being voluntary is crucial, as a voluntary action or a voluntary accepted experience do not increase the Variational Free Energy in the way that an unexpected or involuntary experience does. Computationally, this is a key distinction separating such practices from involuntary experiences that may lead to the onset of post-traumatic conditions.

#### 3.1.7 Emergence and non-linearity

While being a composite, hierarchical system, the Self is not reducible to a set of its components. The functioning of the whole Self is not identical to the functioning of an Autobiographical Self added to Bodily Self and to other components of Selves – these components interact with each other, which creates emergent properties. The Self is therefore a dynamical, non-linear system.^15^ We explore this point in more details in Section 3.2.

#### 3.1.8 A relationship between the Self and models of the outside world

As noted earlier, we have a generative model of the external world (the environment) and a generative model of our body and mind, the experienced components of which we call the Self. In some respects, the second one can be considered as operating at a higher level of the predictive hierarchy than the first one. For example, some subcomponents of our Self may include the inferences about how we infer about the world. An example could be an observation about one’s traits, such as “I am a pessimist” – this is an inference about how we model the world.

Several conclusions follow from this observation. One is that these ‘meta’ parts of the Self tend to operate at the slower time scales than the environment model (Parr et al., 2022). This ‘slowing down’ of temporal scales is the general trend when we move from the periphery to the center of the predictive hierarchy.

Second, the relationship between the generative model of the environment and of the internal milieu is indeed complex. One can imagine some modelling of the world being functional without any meta-inferences about how this process works–the sentience without an awareness of sentience (Frith, 2021). Indeed, while we are often aware of processing information about the world, e.g., via the feeling of mental effort, we are generally ignorant of how this processing works. And at other times, our observations at the meta-level can lead us to noticing an issue in our interactions with the outside world, such as “I am being distractible.”

Together, the meta and the sub models contribute to the hierarchical depth of the generative model and there are many layers of the generative model’s hierarchy (e.g., the representations and re-representations of the Bodily Self, according to Craig, 2002).

#### 3.1.9 Graduality

Each Self component has a gradual nature, the degrees of functioning, as opposed to a binary on/off switch for the entire component. What this implies is that depersonalization is a spectrum, and it is heterogenous; it is not a discrete, homogenous phenomenon. For example, some level of dysfunction in the Autobiographical or the Bodily Self can be considered as a degree of depersonalization.

However, the underlying causes of dissociative experiences do not have to be gradual. An acute onset PTSD can lead to the patient developing dissociative symptoms abruptly and unexpectedly. Similarly, an episode of ketamine intoxication, or an epileptic seizure can abruptly result in dissociative experiences.

#### 3.1.10 Model optimization

Under FEP, a specific optimization of the generative model takes place – the accuracy is maximized while the complexity is minimized (Parr et al., 2022). What this means for the model of the environment and the Self, is that the size of the cognitive light cone described in the TAME framework does not need to exceed what is necessary for the adaptation of a specific agent to its environment. Under FEP, this means that the agent’s goals do not exceed the agent’s preferred states and the behaviors that contribute to visiting such states. From this perspective, there is a certain economy in modeling. A goldfish needs a larger cognitive light cone than a bacterium (Levin, 2022). Humans are capable of having huge cognitive light cones, but they are not restricted to live permanently in a space of long-term plans, a state that would itself be pathological. Arguably, this allows humans to have the greatest adaptability to the most unusual circumstances for which we have no default (innate) strategies.

With that, most people do not operate in a regime of large light cones most of the time at every scale of the brain-mind functioning. For example, paying attention to some immediate task shrinks the cognitive light cone to the near present. One does not daydream while rock climbing, at least not for long. The light cone of an awake and healthy conscious mind may be adaptable to the task at hand. Having a tight temporal focus is not pathological and is sometimes necessary for survival.

### 3.2 Dynamical systems perspective on the health and pathology of the self

The generative models corresponding to each component of the Self operate in various regimes in health and pathology depending on the level of generalized arousal and other circumstances (Tolchinsky, 2023). The system’s change from one regime to another can be described as a phase transition.

**Figure 3:**
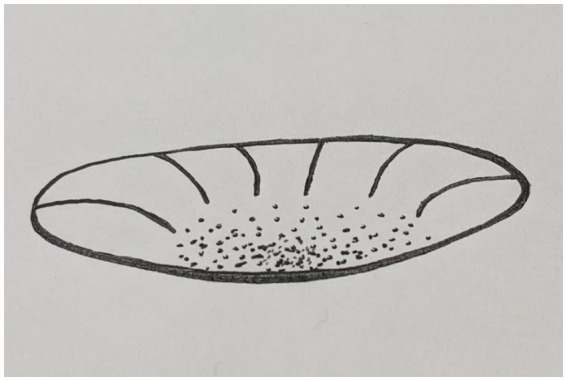
The Self Attractor landscape in health: there is one minimum, corresponding to “I am me,” to which paths in the landscape converge.

When a healthy human subject is awake, we can describe the state space of each Self subcomponent, such as an Autobiographical Self, as operating in a point attractor regime, corresponding to “I am me.” The dissociative experiences in this regime can be mild and benign. The agent returns from these brief fluctuations to the equilibrium point of “I am me.” Put differently, if system is mildly disturbed from the equilibrium point, it will reliably return to it; and if it starts from an initial condition of a mild dissociation it will return to the equilibrium as well. Such slight deviations from the point attractor (the lowest plane of the attractor landscape) can be described as operating in the basin of the point attractor (see Figure 3 for an illustration). The basin consists of all initial conditions that lead to the state of equilibrium “I am me.” In such an attractor landscape a dissociation cannot persist, it is only temporary and mild.

The lowest point of the point attractor corresponds to a minimum of the VFE. A point attractor regime is stable and without external interference of sufficient power, no change in this regime is expected. An acute psychological trauma is one of the examples of such interference, which we think can lead to a phase transition, a period of instability, possibly a chaotic regime of functioning. The repeated and lasting traumatization, such as in C-PTSD can also lead to the destabilization of a point attractor regime of the Self. Then, from a temporarily destabilized, possibly chaotic regime of functioning, the attractor landscape can evolve to various new regimes of some stability, corresponding to the specific post-traumatic presentations.

**Figure 4:**
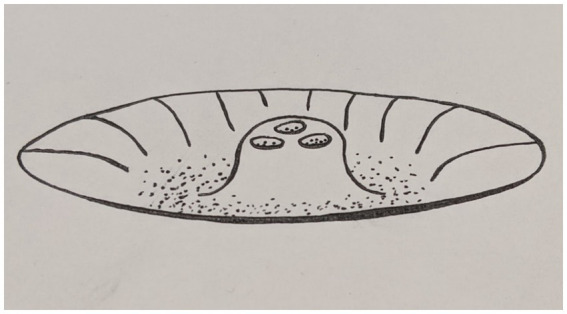
One of the possible attractor landscapes in DID. Here the local minima corresponding to Alters are surrounded by a deeper, global minimum corresponding to a coherent Self.

One of these presentations is the onset of a disorder with chronic and persistent dissociative experiences such as DID or DPDR. As depersonalizations and derealizations become more intense and frequent, a new attractor/repellor landscape corresponding to these experiences evolves. The onset of DID or DPDR is therefore another phase transition, from a transiently chaotic regime to a landscape where relatively stable states corresponding to dissociations are formed. Thus, when a specific pathological condition “takes root,” the system transitions to a multistable mode (Kelso, 2012) with multiple coexisting point attractors. For example, in DID, multiple point attractors may emerge corresponding to each of the alters. See Figure 4 for an illustration.

In DID, when the patient is switching between the alters, we can see an itinerancy, which can be described as a finite set of point attractors, each corresponding to a specific alter in a ‘fragmented’ Autobiographical Self that has lost its cohesion. The patient’s Autobiographical Self being in the state of alter X can be seen as the system moving to the basin of the point attractor “alter X.” The repellor regions in the state space between the point attractors can display chaotic dynamics as expected for the boundary region between the two adjacent point attractors. Accordingly, the switch from one alter to another one is not clearly predictable.

Along with the disruption of cohesion of the Autobiographical Self with the onset of DID, the continuity of time is disrupted as well. The switch from alter X to alter Y disrupts the alter X’s time continuity. Then, effectively, each alter has its own temporary cognitive light cone that is smaller than the light cone of a coherent Self.

Considering the variability in DID, the exact landscape of attractors and repellors may vary depending on the patient’s circumstances and context. On Figure 4 you can see that the local minima of the VFE corresponding to each of the alters is surrounded by a global minimum of VFE corresponding to a coherent Self. This is one of the possible options. An alternative possibility, perhaps at a higher level of pathology, is that the VFE landscape has changed so much that the VFE in the minima of the decoherent Self are lower than VFE of coherent one. Such a condition can be seen as more stable in psychopathology and therefore, more “treatment resistant.”

The attractor landscape corresponding to DID or DPDR may be a relatively stable regime, which is unlikely to change without some form of external interference of sufficient power. Psychotherapy can be such an intervention. In some circumstances, psychotherapy can be augmented with psychodelic treatment or neurostimulation, all of which are various forms of temporarily destabilizing the maladaptive attractor landscape. Then, in treatment, another phase transition can take place to a transiently unstable, possibly chaotic regime with a long-term goal of eventually arriving at a landscape corresponding to healthier functioning.^16^

Then, as recovery from DID or DPDR takes place in psychotherapy, the attractor landscape can change back to the single point attractor regime corresponding to a coherent Self, or at least to an attractor landscape that can be seen as somewhat more coherent–with less local point attractors.

While the phase transition from health to DID is influenced by the external factors that are outside the patient’s control, such as an exposure to a single or multiple traumatic events, psychotherapy can be seen as a controlled, or guided phase transition.

We can describe the specific processes underlying such phase transitions as follows. The nested and embedded Self, containing the Core Self and peripheral Self components, corresponds to a hierarchy of coupled or interconnected attractors. Consistent with FEP, the attractors closer to the Core can be seen as operating at slower time scales. Friston and Kiebel (2009) have suggested that a hierarchical system of coupled attractors can be used to describe the phase transitions, such as a slower attractor possibly controlling the phase transitions of a faster attractor. A similar idea called Orbital Decomposition has been proposed by Guastello et al. (1998) for the hierarchical dynamical systems where one chaotic attractor can be decomposed into a series of limit cycle attractors; then, an element of control can be seen in increasing the relative power of one of these limit cycle attractors.

Based on these ideas by Friston and Guastello, in our model, we propose that the attractor landscape corresponding to the Core Self is influencing the phase transitions of the peripheral Self’s attractors, such as Autobiographical Self. As stated previously, the Core Self is inherently affective. One of the components of any affective state is generalized arousal. It has been proposed elsewhere that the changes in the generalized arousal level can lead to the phase transitions of the entire neocortex from a periodic to chaotic state and back (Tolchinsky, 2023). Similarly, an acute psychological trauma can be described as an affective ‘storm,’ starting from the Core Self increasing the level of generalized arousal, leading to higher energy states in the peripheral Self components, which, in turn, may result in the de-stabilization of the ‘healthy’ point attractor regime in the Autobiographical Self.

An onset of persistent dissociative symptoms in post-trauma can be seen as an adaptation of the peripheral Self components (e.g., Autobiographical Self) into a lower-energy regime, where the affective numbness may take place. This corresponds to the Autobiographical Self “settling down” into a multistable attractor landscape corresponding to DID. Conversely, in an active phase of trauma psychotherapy, the patient is gradually able to tolerate affects to some degree and the Autobiographical Self is moving to a higher energy state, not in an abrupt episode of an affective storm, but in a more gradual fashion. This may be sufficient to cause a controlled de-stabilization of a multistable DID attractor landscape into a temporarily chaotic state, while holding in focus a long-term goal of treatment - to lead the attractor landscape eventually to one corresponding to a coherent Autobiographical Self.

The dynamics described above will influence the temporal depth in the relevant Self components. For example, intact temporal depth is a prerequisite to maintaining the temporal continuity in the Autobiographical Self. Then, a transitional, chaotic phase will be accompanied by a temporary collapse in the Autobiographical temporal depth. An onset of persistent dissociations, corresponding to a multistable attractor regime, will result in a fragmentation of the temporal depth. To summarize, some stability in the attractor landscape is necessary for the maintenance of a healthy temporal depth in each Self component. Conversely, the phase transitions in the attractor landscape and fragmentations, such as an onset of multistabilty will result in the temporal depth collapse.

In the following two sections we will supplement our theoretical model with the clinical practice-based and neurobiological viewpoints on dissociative symptoms and temporal depth.

### 3.3 Clinical practice perspective

Clinicians specializing in dissociations highlight the role of affect in dissociative disorders, more so than memory (Chefetz, 2024, personal communications). What they refer to specifically is the quality of “emotional flatness,” sometimes described by patients as “emotional deadness,” or “numbness.”

This clinical perspective can be integrated with the theoretical models described above. Specifically, the emotional flattening corresponds to an issue with the communications between the Core Self and the peripheral Self’s components via the Core Self’s Markov Blanket. For example, the Autobiographical Self in this regime operates as if it is uninformed by the vital emotional information flows that originate in the Core Self due to the suppression of such information flowing from the Core to the Autobiographical Self across the boundary.

As stated earlier, the Core Self is affective. Each affective system in Panksepp’s framework is complex multi-tiered hierarchy with bottom-up and top-down communications (see Figure 2.3 in Panksepp and Biven, 2012). The emotional flattening corresponds to the predominance of the top-down communications and the downregulation of the bottom-up flows. One of the ways this may be achieved is the prefrontal cortex inhibiting the limbic structures, as described in Section 3.4 below. At the higher levels of consciousness, then, we perceive affects as less intense. In FEP terms this corresponds to the top-down lowering of precision associated with affective prediction error messages.

Furthermore, as noted in Section 3.3, the emotional flatness in DID or DPDR corresponds to the ‘settling down’ of the Autobiographical Self’s attractor landscape to a multistable regime accompanied by a decrease in generalized arousal.

It is necessary to repeat here that emotional flatness represents only one kind of dissociative experience, perhaps a characteristic one for patients with DID or DPDR. A posttraumatic flashback, on the other hand, is accompanied by intense affective activation and it is also a form of dissociation.

Moving on from psychological trauma to other causal factors leading to dissociations, we can consider an extreme example - a complete, involuntary dissociation in humans under general anesthesia. In that state, we have nearly no functional memory beyond basic reflexes (breathing) and no options to choose from. The Self is absent when the individual neurons are dissociated by the anesthetic’s blockade of the bioelectrical connections (Peracchia, 1991; Wentlandt et al., 2006) – the individual cells are fine but the large network capable of grandiose thoughts and goals has temporarily disappeared.

A patient’s recovery from general anesthesia may present a temporarily chaotic regime, which can be seen as a phase transition that in most circumstances leads to the point attractor regime “I am me” in each Self subcomponent – with the same identity the person had prior to being anesthetized. However, in some cases, particularly with elderly patients undergoing long-lasting operations, the patient may experience postoperative delirium (Rengel et al., 2018), dissociative amnesia (Chang et al., 2002), postoperative cognitive dysfunction (POCD, Kotekar et al., 2018). While the exact causes of these conditions are poorly understood, a review by Storrs (2014) suggests that the duration and dynamics of recovery from general anesthesia may be less predictable than was previously thought.

An additional clinical example of a severe dissociation is a seizure, where not only the Autobiographical “I am me” is disrupted, but also the Social Self and possibly other Self components. Similarly to general anesthesia, the necessary resources for the functioning of most, if not all Self models are not operational in this regime. At the level of the scalp EEG dynamics, during a seizure, the neocortex shifts from a chaotic regime in healthy functioning (gamma to high gamma rhythms) to a more orderly regime of slow, high amplitude waves (Tolchinsky, 2023).

Transitions in the sleep/wake cycle can also be seen as potentially leading to a regime change in the Self dynamics. Some theorists suggest that a labile sleep–wake cycle may lead to an intrusion of a dream-like regime into wakefulness, which may lead to dissociative symptoms, including depersonalization (van der Kloet et al., 2012). In addition, the researchers studying derealization report the patients describing this state as being dream-like (van Heugten-van der Kloet and Lynn, 2020).

The process of waking up in the morning can also be seen as a phase transition in the Autobiographical Self, because the orientation to person, place, and time does not happen instantaneously as we wake up (Seth, 2021) and the continuity of the Autobiographical “I am me” does not persist in a linear form as we go through all the transitions in the sleep–wake cycle. Accordingly, individuals with a significant impairment in Autobiographical self, such as Clive Wearing, report their daytime experiences as a series of awakenings, as if they wake up again and again every few minutes (Seth, 2021).

Similarly, substances, such as ketamine can induce a state of consciousness where the Autobiographical or Bodily sense of “I am me” is disrupted, which implies going through a phase transition into a possibly chaotic regime. Notably, patients during the episodes of THC-induced dissociations were found to show features of temporal disintegration (Mathew et al., 1993). Coull et al. (2011) reported disrupted time perception in individuals taking ketamine.

To summarize, the level of consciousness, the regime of consciousness, pathological states, and all the underlying resources necessary for the successful operation of each Self component collectively influence the complex dynamics of the system. A point attractor regime “I am me” in an Autobiographical, Bodily, or other Self components may persist for some time in a healthy, awake human being and such continuity requires the specific state and level of consciousness, as well as the underlying resources, such as a certain level of generalized arousal and functional memory systems.

### 3.4 Neurobiological perspective

#### 3.4.1 Corticolimbic inhibition hypothesis

One of the historically influential neurobiological hypotheses of dissociative disorders is the corticolimbic inhibition (Sierra and Berrios, 1998). According to this model, DPDR is associated with a hyperactivity in the prefrontal cortex (PFC), which results in the PFC exerting increased inhibition of the Anterior Cingulate Cortex (ACC) and the limbic structures, including the amygdala.

Sierra and Berrios (1998) hypothesized that the activation of the right dorsolateral PFC (dlPFC) was accompanied by increased alertness while the reciprocal inhibition of the ACC by the right dlPFC was possibly responsible for the experiences of “mind emptiness” and “indifference to pain.” They further hypothesized that the activation of left PFC regions was responsible for the increased inhibition of the amygdala, which, according to Sierra and Berrios, was responsible for the hypo-emotionality, dampened autonomic output experienced by the patients as the feelings of unreality or detachment.

Subsequent neuroimaging-based studies provided partial support of this hypothesis and suggested that this model would benefit from a revision, such as taking into account the dynamics and contextuality.

For example, Felmingham et al. (2008) who studied PTSD patients with dissociative and non-dissociative presentations via functional magnetic resonance imaging (fMRI), reported that patients with dissociative PTSD showed the activation of the ventral PFC while consciously processing fear-evoking visual stimuli, but not in response to subliminally presented fear-evoking visual stimuli. Felmingham et al. (2008) reported that in response to the subliminally presented fear-evoking visual stimuli patients with dissociative PTSD showed the activation of bilateral amygdala, insula, and left thalamus.

More recently, Medford et al. (2016) used fMRI while presenting visually emotive stimuli to 14 patients with DPDR, as compared to 25 healthy controls. Their results showed decreased activity in the amygdala and hypothalamus in the patient group, coupled with increased activity in the prefrontal regions. However, they did not find differences in the ACC. Additionally, they reported that emotional dampening in the clinical group was associated with reduced activity in the insula, while patients who experienced some improvement in treatment showed increased insula activity on the fMRI. Sierra and Berrios (1998) did not mention the insula as part of their “corticolimbic” inhibition hypothesis.

Lanius et al.’s (2018) paper can be considered as one of the possible revisions of the original corticolimbic inhibition hypothesis. They reviewed recent neuroimaging literature associated with a range of clinical conditions with dissociations – the dissociative subtype of PTSD (PTSD + DS), DID, and dissociations in borderline personality disorder (BDP). Their review supported one of the components of Sierra and Berrios’s (1998) hypothesis–that the emotional dysregulation in patients experiencing a dissociative state can be due to excessive inhibition by the prefrontal regions of the limbic structures, including the amygdala. Lanius et al. referred to this regime as ‘overmodulation’.

With that, they refined Sierra and Berrios’s hypothesis by describing the specific post-traumatic and dissociative regimes and states when the centromedial amygdala (CMA) and basolateral amygdala (BLA) were activated. They also expanded Sierra and Berrios’s (1998) model by including the functioning of the dorsolateral and ventrolateral periaqueductal grey (dl-PAG and vl-PAG respectively), as well as thalamus – making the network of brain regions involved more complex than the network described in the original Sierra and Berrios’s hypothesis.

As another change, Lanius et al. (2018) suggested that while the overmodulation state dominated in patients with PTSD+DS, these patients oscillated between the more prevalent overmodulation and less frequent ‘undermodulation’ regime (excessive activity of the amygdala and hypoactive PFC). In addition, Lanius and colleagues reported that the patients with DID showed varying patterns of activation in different states of functioning. When these patients were observed as in a state with access to the traumatic memories, they showed undermodulation accompanied by thalamic perfusion, as compared to the state of dissociative amnesia.

These observations add dynamics to the original cortico-limbic inhibition model and correspond to what we described in Section 3.3 - an itinerancy around the characteristic states in a disorder corresponding to the specific attractor/repellor landscape of this disorder.

#### 3.4.2 Potential neurobiological correlates of temporal depth collapse

One of the pathways for a temporal depth collapse is dysfunction in various memory systems supporting temporal depth, including episodic memory, working memory, memories related to the functioning of the internal bodily systems (Craig, 2002), etc.

These memory systems can operate at different time scales – an Autobiographical Self that relies on episodic and semantic memory can operate on a scale from minutes to years, while working memory can operate on a scale from seconds to several minutes. Importantly, these memory systems, among their many functions, allow us to integrate experiences. For example, a sequence of episodic memories allows us to have a coherent autobiographical narrative - our personal history (Mitchell, 2023). A continuous functioning of our working memory supports thinking, in which individual thoughts consist of concepts sequenced together and stabilized for some time. We then perceive the sequence of these thoughts as a “train of thought.”

The brain networks mediating the functionality of these memory systems are distributed and complex and describing them in detail is beyond the scope of this paper. However, it is worth mentioning some key components that contribute to these systems.

For example, episodic memory encoding will become permanently dysfunctional with the bilateral damage of the hippocampus (Seth, 2021; Baddeley et al., 2020). Patients with such damage, including Clive Wearing (Seth, 2021) can be described as perpetually depersonalized - they do not have a continuous Autobiographical Self.

A temporary disruption of episodic memory encoding happens during an acute psychological trauma, where a significant elevation of cortisol leads to a temporary dysfunction of the hippocampus that is rich in cortisol receptors (Solms and Turnbull, 2018). Consequently, the episodic memory of the traumatic event might not be not encoded reliably. Thus, an acute trauma may result in the discontinuity in the patient’s Autobiographical Self. A similar autobiographic discontinuity can be observed as a result of an epileptic seizure; however, not only episodic but also other memory systems would be discontinuous around the time of the seizure.

A complete dysfunction of the prefrontal cortex (PFC) and the frontoparietal network would likely render the patient’s working memory dysfunctional (Baddeley et al., 2020). A patient experiencing this state may present as psychotic. It may be hard to assess the severity of the disruption in the coherence of their various Selves, as it would be hard to interview them. The Bodily Self would likely be dysfunctional with damage to the anterior and posterior insula (Craig, 2002).

## 4 Summary

The Self in our model has a nested structure with embedded components and a deep generative model. Stated differently, it is an integrated, multi-layered dynamical system, whose complexity level exceeds that of its components. The size of the Self’s cognitive light cone is one of the measures of its complexity – its temporal depth.

As mentioned in Section 1, the individuals who experienced prolonged, inescapable exposure to highly stressful environments, accompanied by repeated traumatic experiences, tend to develop dissociative disorders. In our model, we can describe this environmental exposure as lasting stress beyond the agent’s ability to manage. Normal cognitive activity - memory access, planning, and redirection of attention under executive control - is disrupted in this regime, leading to a disruption of the experience of linear, integrated time. Such experience is bound to cause the Self’s disintegration – its breakdown into a collection of components, each with a smaller cognitive light cone.

This process will, in our model, be accompanied by a collapse of the temporal depth. The landscape of the agent’s attractors and repellors changes then and the dissociative disorder “takes root.” Then, a sustained effort in psychotherapy is required to help restore the Self’s coherence and continuity – the depth of its generative model and its temporal depth.

In contrast to the inescapable, lasting, overwhelming stress, a single episode of drug use can lead to a temporary dissociation due to the transient disruption of the resources necessary to support the depth of the Self’s generative model – its memory systems. For example, an episode of ketamine use may result in the patient’s working memory disruption, leading to a transient dissociation. We may also experience benign daily dissociations on the border of sleep and wakefulness or during meditation. These transient dissociations do not require sustained therapeutic interventions.

Lasting or temporary, severe or mild, dissociative experiences are accompanied by the collapse of the Self’s temporal depth. In this paper, we have shown from multiple perspectives that temporal depth collapse causes the onset of dissociations, regardless of their etiology.

In the follow-up papers, we will present empirical and clinical data in support of our model and discuss possible therapeutic implications of this model for patients suffering from dissociative disorders.

## Data Availability

The original contributions presented in the study are included in the article/supplementary material, further inquiries can be directed to the corresponding author.

## Footnotes

^1^A collapse to zero (living in the “here and now”) and a collapse to infinity (being unable to differentiate immediate future from long-term future) are both the forms of temporal depth collapse that lead to dissociations. If one cannot distinguish planning for tomorrow from planning for 10 years from now, one cannot plan. Both states involve a collapse of distinctions between the different temporal scales relative to present time, or to current experience. In our paper, the mathematical term “collapse” will correspond to any transition from a single, well-defined, long-term coherent and continuous agent to a shorter, fragmented, discontinuous, or incoherent agent. Thus, a “collapse” can be from well-defined (single) to poorly defined (multiple or fragmented), or from coherent to incoherent.

^2^An example of a successful use of modeling in a biological system is Levin’s (2021) model of cancer. While a focus on just one aspect of cancer in that paper presents a simplification of complex cancer etiology, it allows to make testable predictions, and it is experimentally supported.

^3^While Deane et al. (2020) suggests a possibility of a relationship between temporal depth and dissociations, we make a claim that a collapse of the temporal depth causes the onset of dissociative symptoms. We further suggest that temporal depth collapse can be conceptualized as a common pathway for the onset of dissociative symptoms in circumstances of variable etiology. Finally, we suggest that the focus on restoring temporal depth can be an effective strategy in therapeutic intervensions.

^4^In and of itself, without involuntary, persistent dissociative symptoms, high absorption may be beneficial in a highly demanding or even survival-critical situation (e.g., high-speed driving in traffic). We will discuss this point later in the paper when we describe voluntary versus involuntary dissociations.

^5^We will present a model of a coherent and continuous Self (mental Self, e.g., Self experienced by an agent) in **Section 3** of this paper, which will also include the concepts described elsewhere, e.g., subcomponents of the Self in Chapters 8 and 9 of Seth (2021), a concept of the Core Self in Chapter 11 in Panksepp and Biven (2012), a discussion of temporal depth in Deane et al. (2020). Where our views deviate from these sources, we will articulate the differences as appropriate.

^6^As nearly a tautology, non-linear systems are not additive. What this means is that a collection of components of a non-linear system added together will not produce the entire system. Nonlinear systems cannot be deconstructed into modules without a loss of functioning.

^7^We will refer to the mental/subjective Self as simply Self hereafter. In addition, by Self we will mean the whole Self, including all its components described in this section (e.g., the Core Self, the Autobiographical Self, etc.).

^8^We do not imply philosophical dualism by this statement. We simply draw a distinction between an object and its representation or model. For example, an actual left foot is distinct from the representation of the left foot in the brain.

^9^When we use the spatial terms below in this section, such as “center,” “periphery,” “within,” etc., we use them as metaphors.

^10^The Core Self also contains subcomponents and is thus a composite system in and of itself, it is not a monolithic structure. For the sake of clarity, we will not be describing the internal architecture of the Core Self in this paper.

^11^The VFE in this model is dimensional, each affect corresponds to a specific dimension of the VFE. A prediction error results in the organism’s action, which is an attempt to minimize the specific affective dimension of the VFE.

^12^We chose the term Autobiographical Self for what Seth (2021) describes as the Narrative Self due to episodic (autobiographical) memories being present in rats in the absence of verbal narratives.

^13^One of the reasons we do not list each one is that we attempt to describe the overall architecture, including the hierarchy. A second reason is that some of the peripheral components, such as Seth (2021) “Perspectival Self” are debatable as stable constructs. We disagree with Seth on the utility of describing first person or third person perspectives as specific Self components.

^14^A thesis that collective intelligence participates in human mental functioning, particularly its unconscious aspects, has been proposed before, e.g., Sulis (1997).

^15^This outlook has been proposed before, e.g., Putnam (2016) and we agree with these authors about the non-linearity of the Self.

^16^The therapeutic interventions can only change the free energy landscape. It is not possible to go uphill in an existing, stable VFE landscape. Therefore, in therapeutic work, we may have a goal to erase the current local minima corresponding to the alters in DID or to shift the location of the global minimum.

## Glossary^17^

Generative model: Generative model is a probabilistic model, comprising a likelihood and prior beliefs that specifies how (sensory) consequences are generated by latent (i.e., external) causes, such as hidden states and model parameters
Inference: Inference is the optimization of beliefs by maximizing Bayesian model evidence or minimizing surprise. Approximate Bayesian inference corresponds to minimizing variational free energy
Active inference: Active inference is the minimization of variational free energy through approximate Bayesian inference and active sampling of (sensory) data. This active sampling itself induces posterior beliefs over action, under prior beliefs that action will minimize free energy in the future. This is equivalent to resolving uncertainty with epistemic, information-seeking behavior
Temporal depth: Temporal depth (of counterfactual policies) is the depth of temporal processing during planning as inference
Markov blanket: Markov blanket is the set of variables that mediate all (statistical) interactions between a system and its environment
Cognitive light cone size: Cognitive light cone size is the size or scale of goals any given system can pursue
(Variational) Free Energy: (Variational) Free Energy is a functional of sensory data and posterior beliefs. Free energy scores the surprise of (sensory) data, given posterior beliefs about how they were caused. This furnishes an approximation to Bayesian model evidence, aka marginal likelihood.
Interoceptive: Interoceptive is pertaining to internal (autonomic) states.
Composite system: Composite system is a system that consists of multiple components.
Embedded system: Embedded system is a system that is a part of a larger system. Embedded systems interact with their environments, so cannot be considered isolated.
Nested system: Nested system is a multilayered system with components that in turn contain subcomponents. This process may continue at various scales.
(Fixed) Point attractor: (Fixed) Point attractor is characterized by the state to which a system evolves over time and to which it returns after being perturbed.
Limit cycle attractor: Limit cycle attractor is a closed trajectory in the state space that corresponds to sustained oscillations without decay or growth.
Repellor: Repellor is an area in phase space from which nearby trajectories diverge over time. Unlike an attractor, a repellor is a highly unstable area of phase space.
Attractor landscape: Attractor landscape is a computational model describing a collection of attractors and repellors in phase space (metaphorically, a landscape of valleys and hills) which describes the dynamics of a complex system in a specific regime.
Multistability: Multistability is a system that has multiple coexisting attractors and in which noise is sufficiently strong to cause switching among stable states

## References

[ref1] American Psychiatric Association. (2022). What Are Dissociative Disorders? https://www.psychiatry.org/patients-families/dissociative-disorders/what-are-dissociative-disorders (accessed July 31, 2025).

[ref2] Baddeley A. Eysenck M. W. Anderson M. C. (2020). Memory. London, UK: Routledge.

[ref3] Baluška F. Levin M. (2016). On having no head: cognition throughout biological systems. Front. Psychol. 7:902. doi: 10.3389/fpsyg.2016.00902, PMID: 27445884 PMC4914563

[ref4] Bernstein E. M. Putnam F. W. (1986). Development, reliability, and validity of a dissociation scale. J. Nerv. Ment. Dis. 174, 727–735. doi: 10.1097/00005053-198612000-00004 3783140

[ref5] Blackiston D. Lederer E. Kriegman S. Garnier S. Bongard J. Levin M. (2021). A cellular platform for the development of synthetic living machines. Sci. Robot. 6:eabf1571. doi: 10.1126/scirobotics.abf1571, PMID: 34043553

[ref6] Boyer S. M. Caplan J. E. Edwards L. K. (2022). Trauma-related dissociation and the dissociative disorders. Del. J. Public Health 8, 78–84. doi: 10.32481/djph.2022.05.010, PMID: 35692991 PMC9162402

[ref7] Brand B. L. Schielke H. Schiavone F. Lanius R. A. (2022). Finding solid ground: Overcoming obstacles in trauma treatment. New York, NY: Oxford University Press.

[ref8] Chan J. L. Koval M. J. Womelsdorf T. Lomber S. G. Everling S. (2015). Dorsolateral prefrontal cortex deactivation in monkeys reduces preparatory beta and gamma power in the superior colliculus. Cereb. Cortex 25, 4704–4714. doi: 10.1093/cercor/bhu154, PMID: 25037923 PMC4635915

[ref9] Chang Y. Huang C. H. Wen Y. R. Chen J. Y. Wu G. J. (2002). Dissociative amnesia after general anesthesia--a case report. Acta Anaesthesiol. Sin. 40, 101–104, PMID: 12194389

[ref10] Chefetz R. A. (2015). Intensive psychotherapy for persistent dissociative processes: The fear of feeling real (Norton series on interpersonal neurobiology).. New York, NY: WW Norton & Company. 10.1080/15299732.2016.1205394 27399052

[ref11] Ciaunica A. Seth A. Limanowski J. Hesp C. Friston K. J. (2022). I overthink—therefore I am not: an active inference account of altered sense of self and agency in depersonalisation disorder. Conscious. Cogn. 101:103320. doi: 10.1016/j.concog.2022.103320, PMID: 35490544 PMC9130736

[ref12] Clawson W. P. Levin M. (2022). Endless forms most beautiful 2.0: teleonomy and the bioengineering of chimaeric and synthetic organisms. Biol. J. Linn. Soc. 139, 457–486. doi: 10.1093/biolinnean/blac073

[ref13] Coull J. T. Morgan H. Cambridge V. C. Moore J. W. Giorlando F. Adapa R.. (2011). Ketamine perturbs perception of the flow of time in healthy volunteers. Psychopharmacology 218, 543–556. doi: 10.1007/s00213-011-2346-9, PMID: 21603893 PMC3210361

[ref14] Craig A. D. (2002). How do you feel? Interoception: the sense of the physiological condition of the body. Nat. Rev. Neurosci. 3, 655–666. doi: 10.1038/nrn894, PMID: 12154366

[ref15] Da Costa L. Friston K. Heins C. Pavliotis G. A. (2021). Bayesian mechanics for stationary processes. Proceed. R. Soc. Math. Physical Engin. Sci., 477 (2256) 477:20210518. doi: 10.1098/rspa.2021.0518, PMID: 35153603 PMC8652275

[ref16] Darby R. R. Laganiere S. Pascual-Leone A. Prasad S. Fox M. D. (2016). Finding the imposter: brain connectivity of lesions causing delusional misidentifications. Brain 140, 497–507. doi: 10.1093/brain/aww288, PMID: 28082298 PMC5278302

[ref17] Deane G. Miller M. Wilkinson S. (2020). Losing ourselves: active inference, depersonalization, and meditation. Front. Psychol. 11:539726. doi: 10.3389/fpsyg.2020.539726, PMID: 33250804 PMC7673417

[ref18] Felmingham K. Kemp A. H. Williams L. Falconer E. Olivieri G. Peduto A.. (2008). Dissociative responses to conscious and non-conscious fear impact underlying brain function in post-traumatic stress disorder. Psychol. Med. 38, 1771–1780. doi: 10.1017/s0033291708002742, PMID: 18294420

[ref19] Fischer D. Boes A. Demertzi A. Fischer D. B. Boes A. D. Evrard H. C.. (2016). A human brain network derived from coma-causing brainstem lesions. Neurology 87, 2427–2434. doi: 10.1212/wnl.0000000000003404, PMID: 27815400 PMC5177681

[ref20] Friston K. (2013). Life as we know it. J. R. Soc. Interface 10:20130475. doi: 10.1098/rsif.2013.0475, PMID: 23825119 PMC3730701

[ref21] Friston K. (2018). Am I self-conscious? (or does self-organization entail self-consciousness?). Front. Psychol. 9:579. doi: 10.3389/fpsyg.2018.00579, PMID: 29740369 PMC5928749

[ref22] Friston K. Da Costa L. Sajid N. Heins C. Ueltzhöffer K. Pavliotis G. A.. (2023). The free energy principle made simpler but not too simple. Phys. Rep. 1024, 1–29. doi: 10.1016/j.physrep.2023.07.001

[ref23] Friston K. Kiebel S. (2009). Predictive coding under the free-energy principle. Philos. Transact. R. Soc. B Biol. Sci. 364, 1211–1221. doi: 10.1098/rstb.2008.0300, PMID: 19528002 PMC2666703

[ref24] Friston K. Rigoli F. Ognibene D. Mathys C. Fitzgerald T. Pezzulo G. (2015). Active inference and epistemic value. Cogn. Neurosci. 6, 187–214. doi: 10.1080/17588928.2015.1020053, PMID: 25689102

[ref26] Frith C. D. (2021). The neural basis of consciousness. Psychol. Med. 51, 550–562. doi: 10.1017/s0033291719002204 31481140

[ref27] Graziano M. S. A. Webb T. W. (2015). The attention schema theory: a mechanistic account of subjective awareness. Front. Psychol. 6:500. doi: 10.3389/fpsyg.2015.00500, PMID: 25954242 PMC4407481

[ref28] Guastello S. J. Hyde T. Odak M. (1998). Symbolic dynamic patterns of verbal exchange in a creative problem solving group. Nonlinear Dynamics Psychol. Life Sci. 2, 35–58. doi: 10.1023/a:1022324210882

[ref29] Herman J. L. (2015). Trauma and recovery: The aftermath of violence--from domestic abuse to political terror. New York, NY: Hachette UK.

[ref30] International Society for the Study of Trauma and Dissociation (2011). Guidelines for treating dissociative identity disorder in adults, third revision. J. Trauma Dissociation 12, 115–187. doi: 10.1080/15299732.2011.537247, PMID: 21391103

[ref31] Kelso J. A. S. (2012). Multistability and metastability: understanding dynamic coordination in the brain. Philos. Transact. R. Soc B Biol. Sci. 367, 906–918. doi: 10.1098/rstb.2011.0351, PMID: 22371613 PMC3282307

[ref32] Kotekar N. Shenkar A. Nagaraj R. (2018). Postoperative cognitive dysfunction &ndash; current preventive strategies. Clin. Interv. Aging 13, 2267–2273. doi: 10.2147/cia.s133896 30519008 PMC6233864

[ref33] Lanius R. A. Boyd J. E. McKinnon M. C. Nicholson A. A. Frewen P. Vermetten E.. (2018). A review of the neurobiological basis of trauma-related dissociation and its relation to cannabinoid-and opioid-mediated stress response: a Transdiagnostic, translational approach. Curr. Psychiatry Rep. 20:118. doi: 10.1007/s11920-018-0983-y, PMID: 30402683

[ref34] Levin M. (2019). The computational boundary of a “self”: developmental bioelectricity drives multicellularity and scale-free cognition. Front. Psychol. 10:2688. doi: 10.3389/fpsyg.2019.02688, PMID: 31920779 PMC6923654

[ref35] Levin M. (2021). Bioelectrical approaches to cancer as a problem of the scaling of the cellular self. Prog. Biophys. Mol. Biol. 165, 102–113. doi: 10.1016/j.pbiomolbio.2021.04.007, PMID: 33961843

[ref36] Levin M. (2022). Technological approach to mind everywhere: an experimentally-grounded framework for understanding diverse bodies and minds. Front. Syst. Neurosci. 16:768201. doi: 10.3389/fnsys.2022.768201, PMID: 35401131 PMC8988303

[ref37] Loewenstein R. J. (2018). Dissociation debates: everything you know is wrong. Dialogues Clin. Neurosci. 20, 229–242. doi: 10.31887/dcns.2018.20.3/rloewenstein, PMID: 30581293 PMC6296396

[ref38] Mathew R. J. Wilson W. H. Humphreys D. Lowe J. V. Weithe K. E. (1993). Depersonalization after marijuana smoking. Biol. Psychiatry 33, 431–441. doi: 10.1016/0006-3223(93)90171-9, PMID: 8490070

[ref39] Medford N. Sierra M. Stringaris A. Giampietro V. Brammer M. J. David A. S. (2016). Emotional experience and awareness of self: functional MRI studies of depersonalization disorder. Front. Psychol. 7:432. doi: 10.3389/fpsyg.2016.00432, PMID: 27313548 PMC4890597

[ref40] Melges F. T. Tinklenberg J. R. Hollister L. E. Gillespie H. K. (1970). Temporal disintegration and depersonalization during marihuana intoxication. Arch. Gen. Psychiatry 23, 204–210. doi: 10.1001/archpsyc.1970.01750030012003, PMID: 4916452

[ref41] Metzinger T. (2004). Being No One. Cambridge, MA, USA: MIT/Bradford.

[ref42] Meyer T. Rust N. C. (2018). Single-exposure visual memory judgments are reflected in inferotemporal cortex. eLife 7:e32259. doi: 10.7554/elife.32259, PMID: 29517485 PMC5843407

[ref43] Mitchell K. J. (2023). Free Agents. Princeton, NJ: Princeton University Press.

[ref44] Murphy R. J. (2023). Depersonalization/derealization disorder and neural correlates of trauma-related pathology: a critical review. Innov. Clin. Neurosci. 20, 53–59, PMID: 37122581 PMC10132272

[ref45] Orive G. Taebnia N. Dolatshahi-Pirouz A. (2019). A new era for cyborg science is emerging: the promise of cyborganic beings. Adv. Healthc. Mater. 9:e1901023. doi: 10.1002/adhm.201901023, PMID: 31778037

[ref46] Panksepp J. Biven L. (2012). The archaeology of mind: Neural origins of human emotion. New York, NY: WW Norton & Company.

[ref47] Parr T. Pezzulo G. Friston K. J. (2022). Active inference: The free energy principle in mind, brain, and behavior. Cambridge, MA MIT Press.

[ref48] Peracchia C. (1991). Effects of the anesthetics heptanol, halothane and isoflurane on gap junction conductance in crayfish septate axons: a calcium-and hydrogen-independent phenomenon potentiated by caffeine and theophylline, and inhibited by 4-aminopyridine. J. Membr. Biol. 121, 67–78. doi: 10.1007/bf01870652, PMID: 2051474

[ref49] Pio-Lopez L. (2021). The rise of the biocyborg: synthetic biology, artificial chimerism and human enhancement. New Genetics and Society 40, 599–619. doi: 10.1080/14636778.2021.2007064

[ref50] Putnam F. (2016). The way we are: How states of mind influence our identities, personality, and potential for change. New York, NY: International Psychoanalytic Books.

[ref51] Ramstead M. J. D. (2023) The free energy principle—a Precis. Dialectical Systems. Retrieved from. https://www.dialecticalsystems.eu/contributions/the-free-energy-principle-a-precis/ (accessed July 31, 2025)

[ref52] Rengel K. F. Pandharipande P. P. Hughes C. G. (2018). Postoperative delirium. Presse Med. 47, e53–e64. doi: 10.1016/j.lpm.2018.03.012, PMID: 29680484

[ref53] Rosas F. E. Geiger B. C. Luppi A. I. Seth A. K. Polani D. Gastpar M.. (2024). Software in the natural world: a computational approach to hierarchical emergence. Preprint arxiv:2402.09090v2

[ref55] Rouleau N. Levin M. (2023). The multiple Realizability of sentience in living systems and beyond. Eneuro 10:ENEURO.0375-23.2023. doi: 10.1523/eneuro.0375-23.2023, PMID: 37963652 PMC10646883

[ref56] Seth A. (2021). Being you: A new science of consciousness. New York, NY: Penguin.

[ref57] Shedler J. (2006). Why the scientist–practitioner schism won’t go away. Gen. Psychol. 41, 9–10.

[ref58] Sierra M. (2009). “A history of depersonalization” in In Depersonalization: A New Look at a Neglected Syndrome (Cambridge: Cambridge University Press).

[ref59] Sierra M. Berrios G. E. (1997). Depersonalization: a conceptual history. Hist. Psychiatry 8, 213–229. doi: 10.1177/0957154x9700803002, PMID: 11619439

[ref60] Sierra M. Berrios G. E. (1998). Depersonalization: neurobiological perspectives. Biol. Psychiatry 44, 898–908. doi: 10.1016/s0006-3223(98)00015-8, PMID: 9807645

[ref61] Simeon D. Hwu R. Knutelska M. (2007). Temporal disintegration in depersonalization disorder. J. Trauma Dissociation 8, 11–24. doi: 10.1300/j229v08n01_02, PMID: 17409052

[ref62] Solms M. (2019). The hard problem of consciousness and the free energy principle. Front. Psychol. 9:2714. doi: 10.3389/fpsyg.2018.02714, PMID: 30761057 PMC6363942

[ref63] Solms M. (2021). The hidden spring: A journey to the source of consciousness. Kindle Edn. New York, NY: W.W. Norton & Company.

[ref64] Solms M. Turnbull O. (2018). The brain and the inner world: An introduction to the neuroscience of subjective experience. Abingdon, Oxfordshire, United Kingdom: Routledge.

[ref65] Storrs C. (2014). Hidden Dangers of Going Under. Sci. Am. 310, 34–35. doi: 10.1038/scientificamerican0414-34, PMID: 24712120

[ref66] Sulis W. (1997). Collective intelligence as a model for the unconscious. Psychol. Perspect. 35, 64–91. doi: 10.1080/00332929708403312

[ref67] Tellegen A. Atkinson G. (1974). Openness to absorbing and self-altering experiences (“absorption”), a trait related to hypnotic susceptibility. J. Abnorm. Psychol. 83, 268–277. doi: 10.1037/h0036681, PMID: 4844914

[ref68] Tognoli E. Kelso J. S. (2014). The metastable brain. Neuron 81, 35–48. doi: 10.1016/j.neuron.2013.12.022 24411730 PMC3997258

[ref70] Tolchinsky A. (2023). A case for chaos theory inclusion in neuropsychoanalytic modeling. Neuropsychoanalysis 25, 43–52. doi: 10.1080/15294145.2023.2191983

[ref71] Vallacher R. R. Van Geert P. Nowak A. (2015). The intrinsic dynamics of psychological process. Curr. Dir. Psychol. Sci. 24, 58–64. doi: 10.1177/0963721414551571

[ref72] van der Kloet D. Merckelbach H. Giesbrecht T. Lynn S. J. (2012). Fragmented Sleep, Fragmented Mind. Perspect. Psychol. Sci. 7, 159–175. doi: 10.1177/1745691612437597, PMID: 26168441

[ref73] van Heugten-van der Kloet D. Lynn S. J. (2020). Dreams and dissociation—commonalities as a basis for future research and clinical innovations. Front. Psychol. 11:745. doi: 10.3389/fpsyg.2020.00745, PMID: 32390913 PMC7189023

[ref74] Vonderlin R. Kleindienst N. Alpers G. W. Bohus M. Lyssenko L. Schmahl C. (2018). Dissociation in victims of childhood abuse or neglect: a meta-analytic review. Psychol. Med. 48, 2467–2476. doi: 10.1017/s0033291718000740, PMID: 29631646

[ref75] Wentlandt K. Samoilova M. Carlen P. L. Beheiry H. E. (2006). General anesthetics inhibit gap junction communication in cultured organotypic hippocampal slices. Anesth. Analg. 102, 1692–1698. doi: 10.1213/01.ane.0000202472.41103.78, PMID: 16717311

[ref76] Yonelinas A. P. Ramey M. M. Riddell C. Kahana M. J. Wagner A. D. (2022). “Recognition memory: The role of recollection and familiarity” in The Oxford handbook of human memory. Oxford, UK: Oxford University Press.

